# Urban and semi-urban mosquitoes of Mexico City: A risk for endemic mosquito-borne disease transmission

**DOI:** 10.1371/journal.pone.0212987

**Published:** 2019-03-06

**Authors:** Eduardo Dávalos-Becerril, Fabián Correa-Morales, Cassandra González-Acosta, Rene Santos-Luna, Jorge Peralta-Rodríguez, Crescencio Pérez-Rentería, José Ordoñez-Álvarez, Herón Huerta, Mariana Carmona-Perez, José Alberto Díaz-Quiñonez, María Dolores Mejía-Guevara, Gustavo Sánchez-Tejeda, Pablo Kuri-Morales, Jesús Felipe González-Roldán, Miguel Moreno-García

**Affiliations:** 1 Centro Nacional de Programas Preventivos y Control de Enfermedades, Mexico City, Mexico; 2 Instituto Nacional de Salud Pública, Cuernavaca, Morelos, México; 3 Unidad de Bioensayos-Centro Regional de Control de Vectores Panchimalco-Servicios de Salud de Morelos, Morelos, Mexico; 4 Instituto de Diagnóstico y Referencia Epidemiológicos “Dr. Manuel Martínez Báez”, México City, México; 5 Servicios de Salud de Morelos, Cuernavaca, Morelos, Mexico; 6 Secretaría de Salud de la Ciudad de Mexico, Mexico City, Mexico; 7 Secretaría de Salud, México City, México; Texas A&M University College Station, UNITED STATES

## Abstract

Since past century, vector-borne diseases have been a major public health concern in several states of Mexico. However, Mexico City continues to be free of endemic mosquito-borne viral diseases. The city is the most important politic and economic state of Mexico and one of the most important city of Latin America. Its subtropical highland climate and high elevation (2240 masl) had historically made the occurrence of *Aedes* species unlikely. However, the presence of other potential disease vectors (*Culex* spp, *Culiseta* spp), and the current intermittent introductions of *Aedes aegypti*, have revealed that control programs must adopt routine vector surveillance in the city. In this study, we provide an updated species list from a five-years of vector surveillance performed in Mexico City. A total of 18,553 mosquito larvae were collected. Twenty-two species from genus *Culex*, *Aedes*, *Culiseta*, *Anopheles*, *Lutzia* and *Uranotaenia* were observed. Nine new mosquito records for the city were found. *Ae*. *albopictus* was recorded for the first time in Mexico City. Interestingly, a new record, *Ae*. *epactius* was the most frequent species reported. C*x*. *pipiens quinquefasciatus* exhibited the highest number of individuals collected. We detected six areas which harbor the highest mosquito species records in the city. Cemeteries included 68.9% of our collection sites. Temporarily ponds showed the highest species diversity. We detected an increasing presence of *Ae*. *aegypti*, which was detected for three consecutive years (2015–2017), predominantly in the warmer microclimates of the city. We found a possible correlation between increasing temperature and *Ae*. *aegypti* and *Ae*. *albopictus* expanding range. This study provides a starting point for developing strategies related to environmental management for mosquito control. The promotion of mosquito control practices through community participation, mass media and education programmes in schools should be introduced in the city.

## Introduction

Between 225–247 species of mosquitoes from 20 genera are known to occur in the 32 states of México [1,2]. However, throughout the country, *Aedes aegypti* is the main vector of mosquito-borne viral diseases. Although the presence of *Aedes albopictus* has also been reported in several states [3], its impact as a relevant vector in Mexico is yet unknown. Interestingly, only Mexico City and the state of Tlaxcala remain without the endemic presence of *Aedes spp* mosquitoes. Thus far, both states continue to be free of endemic mosquito-borne viral diseases.

**Fig 1:**
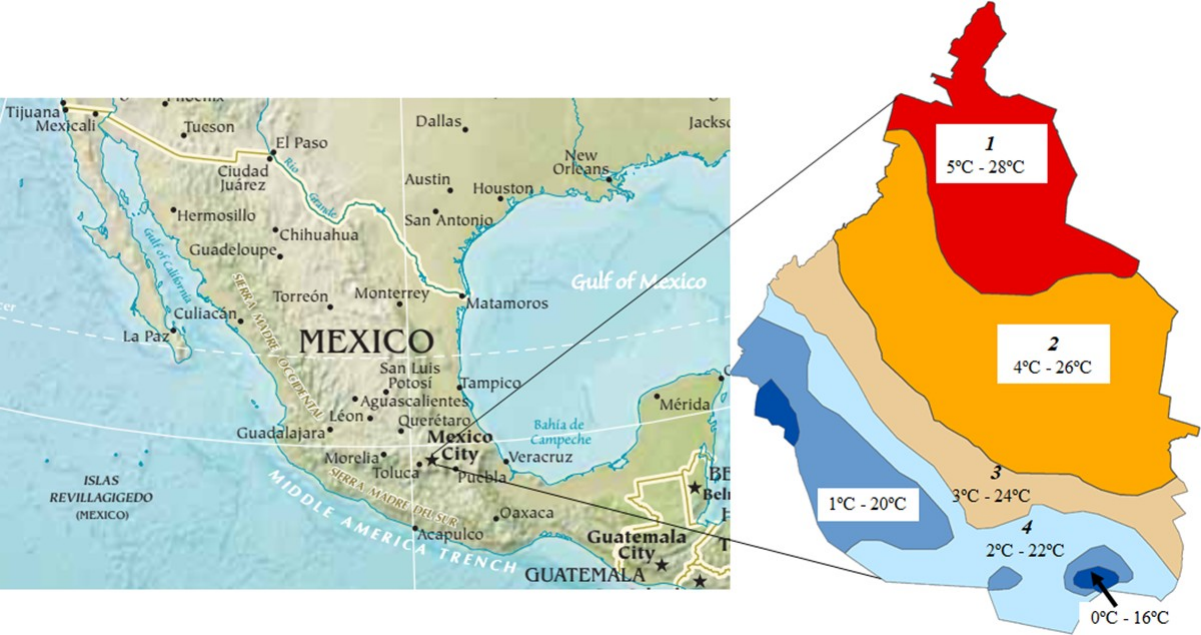
Mexico city location. Lowest and highest average annual temperatures in the city are shown. Map modified from https://www.cia.gov/library/publications/the-world-factbook/attachments/docs/original/north_america.pdf?1528326233.

México City (19°25′57.85″N, 99°07′59.71″W) is the capital of Mexico and is the most important center for all types of financial, cultural and politic activities of the country. Is a destination and point of departure of most international flights, including those which use the city as a stopover from Central and South America, the US and Europe. It is the most urbanized and populous city of the country (8,985,339 people) with a relative small area (1485.5 km^2^) [4]. The city is located at the Basin of Mexico (Fig 1), in an elevated valley at an altitude of about 2,240 m [5]. The city is surrounded by the Trans-Mexican volcanic belt. It has an average annual temperature range of -2^o^ C to 28^o^ C with humid/subhumid/dry and cold/template climates, associated with seasonal rains in the summer and drier and colder weather in the winter (average annual rainfall: 600 to 1200 mm) (sources: Comisión Nacional del Agua-México and Universidad Nacional Autónoma de México). At the south, the city is bordered by the Ajusco-Chichinautzin mountain range and suburban areas including pine-oak forests and an agricultural lowland. The northern and eastern (drier) regions of the city are also bordered by mountain systems covered by grassland. (http://www.cuentame.inegi.org.mx/monografias/informacion/df/default.aspx?tema=me&e=09). These altitude and climate characteristics of the region have historically made the occurrence of *Aedes spp*. unlikely. However, a recent survey detected the intermittent presence of *Ae*. *aegpyti* larvae in the city [4]. Furthermore, previous surveys [4,6–10] had reported the presence of other competent arbovirus vectors including *Culex*, *Culiseta* and *Ochlerotatus* species, indicating that vector surveillance and control programs should adopt a routine monitoring and surveillance scheme in the city.

*Ae*. *aegypti* is a successful invasive species. Characteristics such as desiccation-resistant eggs [11] and exploitation of urban habitats [12] contribute to the likelihood of *Aedes* mosquitoes becoming established in non-native areas. The expansion of the range of *Ae*. *aegypti* into highland areas around the world might be facilitated by climate change due to rapid and extensive urbanization [13]. The expansion may negatively impact resident species because of interspecific competition (e.g. [14]). Likewise, competition could be altering species composition and may have effects on the vector potential of other medically important species (*Aedes* and non-*Aedes*) (e.g. [15]). Information on the diversity of mosquitoes is important to understand the effects of invasive species on native species.

In the present study, we provide an updated species list from five-years of mosquito surveillance performed in México City. Mosquito larvae were collected in urban areas (with high population density, public transportation and minimal green areas [8]) and semi-urban areas (a mix of buildings and green areas with sufficient public transportation but where people still partially maintain agricultural activities [8]). We discuss the potential establishment of *Ae*. *aegypti* and *Ae*. *albopictus* its impact on both the native mosquito species and on public health. Finally, we show a possible correlation between increasing temperatures in the city and the recent presence of *Ae*. *aegypti* and *Ae*. *albopictus*.

## Methods

Larva collections came from 37 surveys performed across Mexico City over the period of 2012–2016. A total of 163 sites were inspected. Fourth instar larval samples were taken from a variety of habitats (natural and artificial) including: 1) cemeteries, 2) wetlands: non-tidal, semi-permanent wet areas, 3) municipal rain collectors: >25 m^2^ cement water containers, 4) natural lakes, 5) ponds: permanent bodies of water (natural or artificial) located in public parks or ecological preserves, 6) temporarily pools: seasonal small (< 5m^2^) bodies of water (natural or artificial) in parks and peri-domestic cemented tanks, 7) streams: natural or human-created shallow tributaries and low flow bodies of water 8) transportation canals: a 100-km network of 10–20 m width and 1–3 m deep canals, commonly used for tourism and food transportation, 8) Olympic canoe canals: a 27 ha and 2m deep artificial canoe sprint and rowing venue.

For larval collections, transfer pipettes and/or 500-ml plastic dippers were used. Collected larvae were placed into bottles containing 96% alcohol. Georeferenced coordinates for each collection site were obtained using a handheld GPS. Collected larvae were then transported to the laboratory. Larvae were counted and identified to species under 50x magnification of a stereo-microscope. Taxonomic identification was carried out using morphological identification keys [16], [17], [18].

**Fig 2:**
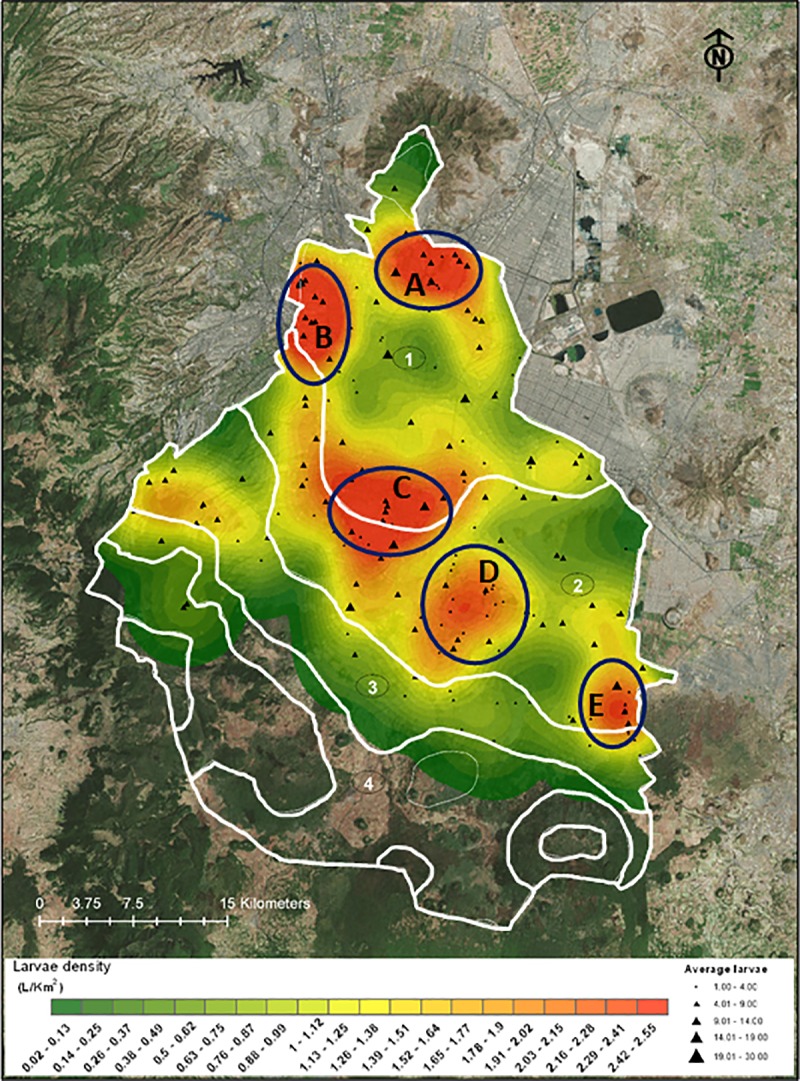
Collecting sites and hotspots. Six cluster of species (clusters A, B, C, D and E) are shown. Numbers indicate microclimates: 1) 5°C—28°C, 2) 4°C—26°C, 3) 3°C—24°C and 4) 2°C—22°C. Larvae density (larvae/km2; green- red areas) and average collected larvae (black triangles) are shown.

The Jaccard similarity coefficient [19] was used to determine similarity in species composition among four temperature gradients where species occurred: microclimate one: 5°C—28°C, microclimate two: 4°C—26°C, microclimate three: 3°C—24°C, microclimate four: 2°C—22° (Figs 1 and 2). Values close to 0 indicate that the microclimates have no species in common and values close to 1 indicate that each species that occurs in one microclimate also occurs in the others.

For *Ae*. *aegypti* and *Ae*. *albopictus*, collections (2015–2017) were performed using artificial ovitraps. The ovitraps used were 1-liter dark plastic cups, filled with tap water and lined with a strip of pellon paper along the water margin. In 2015–2016, eggs collected weekly from 2,253 ovitraps were transported and hatched. Fourth instar larvae were identified using a morphological identification key [10]. For the 2017 collection, eggs were hatched, and larvae were reared to adults and identified to species after emergence. Larvae were maintained at 28°C ± 2°C with 70–80% relative humidity and a photoperiod of 12:12 (L: D) h.

**Fig 3:**
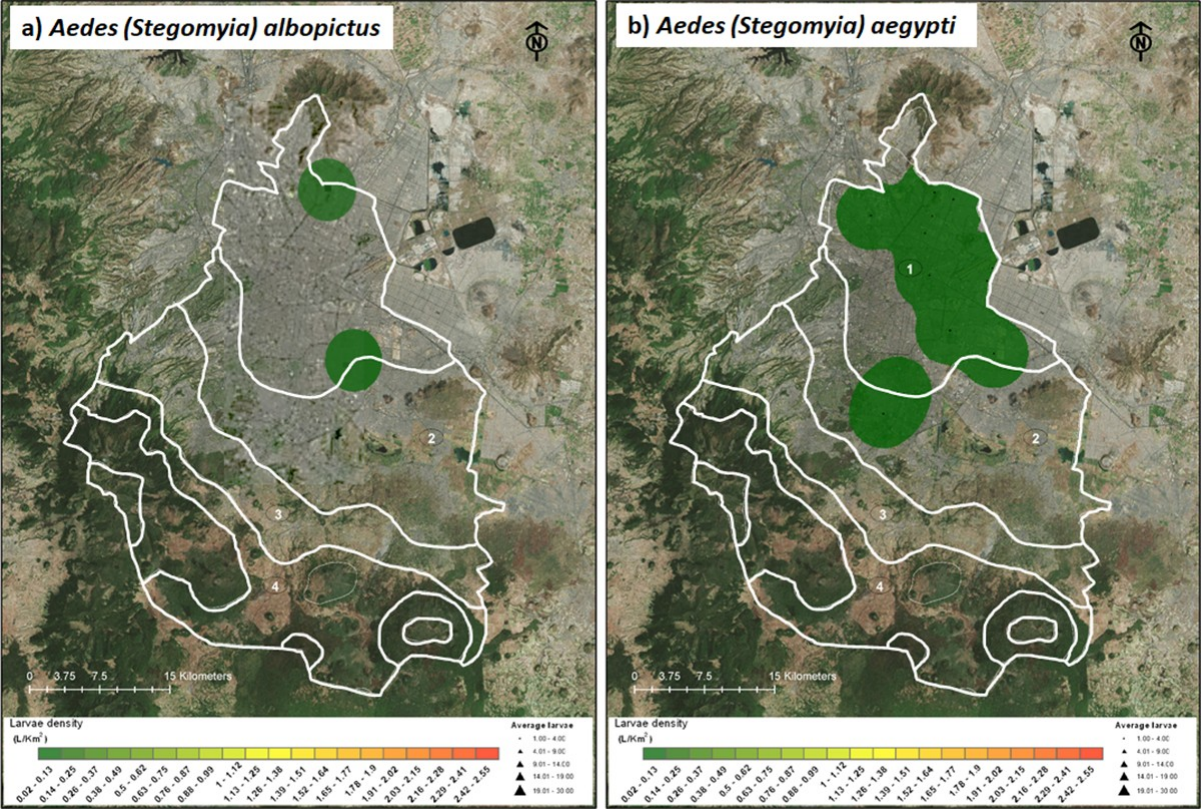
Distribution of *Aedes aegypti* and *Aedes albopictus* of Mexico City eggs from ovitraps collected in the present study.

As a first step to detect the potential risk of *Ae*. *aegypti* introduction and establishment, the possible effects of climate change were assessed. *Ae*. *aegypti* distribution was mapped using Mexico City’s climate projection (modified from [20]). This projection reflects the past and current/future urban micro-climate changes arising from urban expansion and other physical characteristics, waste heat release, and regional climate factors. Maps visualizations (Figs 2 and 3, S2.1–S2.6 Fig) were performed using the ArcGIS version 10. The GPS information of every number of larvae of each specie was input. To make density maps (average larvae per collecting site; larvae/km^2^) the function “Spatial Analyst Tools—Density—Kernel Density” in ArcGIS 10.

All collected specimens, were hatched, reared, identified and deposited in the Laboratory of Entomology at the Instituto de Diagnóstico y Referencia Epidemiológicos (Institute of Epidemiological Diagnosis and Reference, Mexico City, Mexico) and Unidad de Bioensayo-Centro Regional de Control de Vectores (Bioassay Unit-Regional Center of Vector Control Panchimalco, Morelos, Mexico).

### Ethics statement

Note that: no specific permits were required for the described field studies; the location is not privately-owned; the field studies did not involve endangered or protected species.

### Data availability statement

Data used in this study can be found at: dx.doi.org/10.6084/m9.figshare.7400018. Field larvae and ovitrap collection guides can be found at http://www.cenaprece.salud.gob.mx/programas/interior/vectores/descargas/pdf/guia_colecta_entomologica_InDRE.pdf and https://www.gob.mx/cms/uploads/attachment/file/37865/guia_vigilancia_entomologica_ovitrampas.pdf

## Results

A total of 18,546 mosquito larvae belonging to twenty-two species were collected in urban and semi-urban areas of Mexico City (Fig 2, S1 Fig), including the following genus: *Culex* (thirteen species), *Aedes* (one specie;), *Culiseta* (two species), *Anopheles* (two species), *Lutzia* (one species) and *Uranotaenia* (two species) (Table 1, Fig 3 and S2–S7 Figs). *Ae*. *aegypti* and *Ae*. *albopictus* were collected as eggs only. Nine new mosquito records for the city were found: *Anopheles punctipennis*, *Aedes albopictus*, *Aedes epactius*, *Aedes scapularis*, *Culex bidens*, *Culex pinarocampa*, *Lutzia bigoti*, *Culex erraticus*, and *Uranotaenia lowii* (Tables 1 and 2).

**Table 1:** Percentage occurrence (from 163 collecting sites) and percentage of the total number of individuals (out of 18,546 collected larvae) of each mosquito species collected.

*Species*	*% occurrence*	*% individuals*
Aedes (Ochlerotatus) epactius*	90.8	27.40
*Culex (Culex) stigmatosoma*	78.53	16.10
*Culiseta (Culiseta) particeps*	67.48	14.34
*Culex (Culex) pipiens quinquefasciatus*	63.8	28.84
Culex (Culex) pinarocampa*	29.45	3.70
*Culiseta (Culiseta) inornata*	23.93	2.18
*Culex (Culex) thriambus*	18.4	0.65
*Culex (Culex) tarsalis*	16.65	1.02
*Culex (Culex) salinarius*	15.95	1.67
*Culex (Neoculex) arizonensis*	9.82	1.06
*Anopheles (Anopheles) aztecus*	6.75	1.27
*Culex (Culex) erythrothorax*	4.29	0.08
Culex (Culex) bidens*	3.68	0.06
*Culex (Neoculex) apicalis*	3.68	1.34
Lutzia (Lutzia) bigoti*	2.45	0.03
Culex (Melanoconion) erraticus*	2.45	0.09
*Uranotaenia (Uranotaenia) sapphirina*	2.45	0.04
Anopheles (Anopheles) punctipennis*	1.84	0.02
*Culex (Culex) coronator*	1.84	0.10
Aedes (Ochlerotatus) scapularis*	0.61	0.01
*Culex (Culex) restuans*	0.61	0.01
Uranotaenia (Uranotaenia) lowii*	0.61	0.01

*Species marked with asterisks are new records for Mexico City.

**Table 2:** Updated list of urban and semi urban mosquitoes collected in Mexico city.

*Species*	*Temperature/climate range in Mexico City*	*Reported by*	*Pathogens detected elsewhere (but see* *†)*
*Aedes (Stegomyia) albopictus* (Skuse 1894)*	5°C—28°C; subhumid/template/dry	Present study (new record)	WNV, DV, CVV, ZIKV, CHIKV, JEV, EEEV, POTV, USUV, TENV, KEYV, LaCrosse, JCV [21, 22,23]
*Aedes (Stegomyia) aegypti* (Linnaeus 1762)*	5°C—28°C, 4°C—26°C; subhumid/template/dry	Present study, Kuri- Morales et al. (2017)	DV, YF, ZIKV, CHIKV, RR, WNV, DNV, CPV [21, 24, 25]
***Aedes (Ochlerotatus) epactius* (Dyar & Knab 1908)**	5°C—28°C, 4°C—26°C, 3°C—24°C; subhumid/template/dry	Present study (new record)	WNV [21]
***Aedes (Ochlerotatus) scapularis* (Rondani 1848)**	4°C—26°C; subhumid/template	Present study (new record)	YF, REV, CVV, MV, IV, MyV, VEE [24,26,27]
*Aedes (Ochlerotatus) trivittatus* (Coquillett 1902)	No data	Diaz-Najera & Vargas (1973), Heinemann & Belkin (1977), Ibañez- Bernal & Martinez- Campos (1994)	WNV, TV [21,24]
*Anopheles (Anopheles) aztecus* (Hoffman 1935)	4°C—26°C; subhumid/template	Present study, Heinemann & Belkin (1977), Ibañez- Bernal & Martinez- Campos (1994)	*Plasmodium gonderi* [28,29]
*Anopheles (Anopheles) pseudopunctipennis* (Theobald 1901)	No data	Heinemann & Belkin (1977)	*Plasmodium spp* [24]
***Anopheles (Anopheles) punctipennis* (Say 1823)**	4°C—26°C; subhumid/template	Present study (new record)	*Plasmodium spp*, WNV [21,24]
***Culex (Culex) bidens* (Dyar 1922)**	5°C—28°C, 4°C—26°C; subhumid/template/dry	Present study (new record)	VEE [30]
*Culex (Culex) coronator* (Dyar and Knab 1906)	4°C—26°C; subhumid/template	Present study, Ibañez-Bernal & Martinez-Campos (1994)	WNV, ZIKV [21,25]
*Culex (Culex) erythrothorax* (Dyar 1907)	4°C—26°C; subhumid/template	Present study, Martinez-Palacios (1952), Diaz-Najera & Vargas (1973)	WNV [21]
*Culex (Culex) peus* (Speiser 1904) ^**⁂**^	No data	Diaz-Najera & Vargas (1973), Heinemann & Belkin (1977)	—
***Culex (Culex) pinarocampa* (Dyar & Knab 1908)**	5°C—28°C, 4°C—26°C, 3°C—24°C; subhumid/template/dry	Present study (new record)	—
*Culex (Culex) pipiens quinquefasciatus* (Say 1823)	5°C—28°C, 4°C—26°C, 3°C—24°C; subhumid/template/dry	Present study, Diaz-Najera & Vargas (1973), Heinemann & Belkin (1977), Ibañez- Bernal & Martinez- Campos (1994), Diaz- Badillo et al. (2011)	WNV, SLE, NPV, CPV, †**ZIKV in Mexico City** [21,24,25,31]
*Culex (Culex) pipiens pipiens* (Linnaeus 1758)	4°C—26°C, 3°C—24°C, 2°C—22°C; humid/subhumid/template	Diaz- Badillo et al. (2011)	WNV, SLE, NVP [21,24,31]
*Culex (Culex) restuans* (Theobald 1901)	5°C—28°C; dry	Present study, Heinemann & Belkin (1977), Ibañez- Bernal & Martinez- Campos (1994)	WNV, NVP [21,24]
*Culex (Culex) salinarius* (Coquillett 1904)	5°C—28°C, 4°C—26°C; subhumid/template/dry	Present study, Diaz-Najera & Vargas (1973), Ibañez-Bernal & Martinez-Campos (1994)	NVP, DNV [31]
*Culex (Culex) stigmatosoma* (Dyar 1907)	5°C—28°C, 4°C—26°C, 3°C—24°C; subhumid/template/dry	Present study, Martinez-Palacios (1952), Ibañez-Bernal & Martinez-Campos (1994)	SLE [24]
*Culex (Culex) tarsalis* (Coquillett 1896)	5°C—28°C, 4°C—26°C, 3°C—24°C; subhumid/template/dry	Present study, Martinez-Palacios (1952), Diaz-Najera & Vargas (1973), Heinemann & Belkin (1977), Ibañez- Bernal & Martinez- Campos (1994), Diaz- Badillo et al. (2011)	WNV, WEE, DNV, SLE, ZIKV [21,24,25,31,32]
*Culex (Culex) thriambus* (Dyar 1921) ^**⁂**^	5°C—28°C, 4°C—26°C, 3°C—24°C; subhumid/template/dry	Present study, Diaz-Najera & Vargas (1973), Ibañez- Bernal & Martinez- Campos (1994)	WNV, SLE [21,33]
***Culex (Melanoconion) erraticus* (Dyar & Knab 1906)**	4°C—26°C, 3°C—24°C; subhumid/template	Present study (new record)	WNV, VEE [21,34]
*Culex (Neoculex) apicalis* (Adams 1903)	4°C—26°C; subhumid/template	Present study, Diaz-Najera & Vargas (1973)	WNV [21]
*Culex (Neoculex) arizonensis* (Bohart 1948)	4°C—26°C, 3°C—24°C, 2°C—22°C; humid/subhumid/template/dry	Present study, Martinez-Palacios (1952), Diaz-Najera & Vargas (1973), Heinemann & Belkin (1977)	—
*Culiseta (Culiseta) dugesi* (Dyar and Knab, 1906)^◆^	No data	Heinemann & Belkin (1977)	—
*Culiseta (Culiseta) inornata* (Williston 1893)	5°C—28°C, 4°C—26°C, 3°C—24°C; subhumid/template/dry	Present study, Diaz-Najera & Vargas (1973), Heinemann & Belkin (1977), Ibañez- Bernal & Martinez- Campos (1994)	La Crosse, MV, WNV [21,24]
*Culiseta (Culiseta) particeps* (Adams 1903) ^◆^	5°C—28°C, 4°C—26°C, 3°C—24°C, 2°C—22°C; humid/subhumid/template/dry	Present study, Diaz-Najera & Vargas (1973), Ibañez-Bernal & Martinez-Campos (1994)	WNV [21]
***Lutzia (Lutzia) bigoti*** (Bellardi 1862)	4°C—26°C, 3°C—24°C; subhumid/template/	Present study (new record)	—
*Psorophora (Janthinosoma) cyanescens* (Coquillett 1902)	No data	Diaz-Najera & Vargas (1973), Ibañez- Bernal & Martinez- Campos (1994)	Flavivirus, VEE [35,36]
***Uranotaenia (Uranotaenia) lowii* (Theobald 1901)**	4°C—26°C; subhumid/template	Present study (new record)	WNV [37]
*Uranotaenia (Uranotaenia) sapphirina (Osten Sacken 1868)*	4°C—26°C; subhumid/template	Present study, Diaz-Najera & Vargas (1973), Ibañez-Bernal & Martinez-Campos (1994)	WNV, NPV, CPV [21,31]

*Ae. *aegypti* and *Ae*. *albopictus* were collected using ovitraps.

⁂The name *Cx*. *peus* has been updated to *Cx*. *thriambus*.

◆*Cs*. *particeps i*s the contemporary preferred name of *Cs*. *dugesi*.

**†**Determined at the Instituto de Diagnóstico y Referencia Epidemiológicos (Institute of Epidemiological Diagnosis and Reference, Mexico). Listed pathogens were intentionally limited to arbovirus and Plasmodium spp. CHIKV = Chikungunya virus, CPV = Cytoplasmic Polyhedrosis virus, CVV = Cache Valley virus, DNV = Densovirus, DV = Dengue virus, IV = Ilheus virus, EEEV = Eastern Equine Encephalitis virus, JCV = Jamestown Canyon virus, JEV = Japanese encephalitis virus KEYV = Keystone virus, MV = Melao virus, MyV = Mayaro virus, NPV = Nucleopolyhedrovirus, POTV = Potosi virus, REV = Rocio Encephalitis virus, RR = Ross River virus, SLE = Saint Louis Encephalitis, TENV = Tensaw virus, TV = Trivittatus virus, VEE = Venezuelan Equine Encephalitis virus, WEE = Western Equine Encephalomyelitis virus, USUV = Usuto virus, WNV = West Nile virus, YF = Yellow Fever, ZIKV = Zika virus.

*Aedes epactius*, *Culex pipiens quinquefasciatus*, *Culex stigmatosoma*, *Culiseta particeps and Culex pinarocampa* were the most frequent species collected in the city (occurring in 63–90% of collecting sites, Table 1). *Ae*. *epactius* was the most frequent species collected in the city (particularly in cemeteries) (S2 Fig) (Table 1). *Aedes scapularis*, *Cx*. *restuans* and *Ur*. *lowii* were the least frequent and numerous species (Table 1; S2, S4 and S6 Figs). Five species previously recorded in the city were not found during our surveys (see Table 2). *Cx*. *peus* can be excluded from previous records since it has been synonymized with *Cx*. *thriambus* [38]. Likewise, *Cs*. *particeps* is the contemporary preferred synonym of *Cs*. *dugesi* [1].

The taxonomical status of *Cx pipiens* and *Cx*. *p*. *quinquefaciatus* has not resulted in a consensus. Ortega-Morales et al. (2015), stated that true *Cx*. *pipiens* occurs in the northern United States and Canada and in Argentina and Uruguay. However, in Mexico City, the presence of *Cx*. *p*. *quinquefasciatus*, *Cx*. *pipiens* and hybrids has been previously detected by molecular methods [8]. In the present study specimens were classified as *Cx*. *p*. *quinquefasciatus*.

Fourteen species, including *Ae*. *aegypti* and *Ae*. *albopictus*, were reported in microclimate one, twenty-two (including *Ae*. *aegypti* and *Ae*. *albopictus*) in microclimate two, eleven in microclimate three and two in microclimate four (Table 2, Figs 2 and 3, S2–S7 Figs). *Cx*. *restuans* was only found in microclimate one (S4 Fig). Eight species were only found in microclimate two: *Ae*. *scapularis*, *An*. *aztecus*, *An*. *Punctipennis* (S2 Fig), *Cx*. *coronator*, *Cx*. *Erythrothorax* (S3 Fig), *Cx*. *Apicalis* (S5 Fig) *and Ur*. *sapphirina* (S7 Fig), while *Cs*. *particeps* was the only species distributed across the four microclimates (S6 Fig). The Jaccard similarity coefficients were: 0.31 between microclimate one and two; 0.33 between one and three; 0.31 between two and three; 0.15 between three and four; 0.08 between two and four; 0.06 between one and four.

We detected six clusters which harbored the greatest diversity of species in the city (Fig 2). The first cluster was located northwest of the city (spot A, Fig 2). This area corresponds to a set of cemeteries (Cementerio Español, San Isidro, Monte Sinai, Americano and Aleman) and a 77.4 ha park (Parque Ex Refineria 18 de Marzo Centenario). In 1991, this former refinery became a public park with artificial ponds and temporarily pools. The second cluster corresponds to the Basilica of Our Lady of Guadalupe and the foothills of “El Tepeyac” Hill National Park, on the north side of the city (spot B, Fig 2). The third cluster (spot C, Fig 2), corresponds to an urban developments called “Ciudad Jardin/Churubusco/Coyaacan” area (Spot D, Fig 2), which consists of buildings, parks and houses surrounded by small green areas. The fourth cluster and more extensive cluster (mid-south of the city, spot D, Fig 2), corresponds to the canals of the Ecological Park of “Xochimilco” (215 ha), along with several artificial agricultural plots called “chinampas”. This is also a tourist area with water-traffic corridors, wetlands, natural lakes, streams and a canoe canal. The area also contains a mix of urban and semi-urban settlements. Four species were only found in this area (*An*. *aztecus*, *An*. *punctipennis*, *Ur*. *lowii* and *Ur*. *sapphirina*) (S2, S6 and S7 Figs). Cluster six was located in the southeast (spot E, Fig 2), and corresponds to the “Milpa Alta” borough. It is a relatively new semi-urban area with surface water restricted to small springs, streams and several city rain collectors. It is part of the Chichinautzin biological corridor (which also includes the States of Mexico and Morelos).

Cemeteries included 68.9% of our collecting sites, and a great number of species were recorded in this habitat (Table 3). However, cemeteries did not present the highest species diversity; temporary ponds showed the highest diversity (15 species). Cemeteries and water corridors harbored 14 species each and 10 species each were recorded from city rain collectors and wetlands. Ponds, natural lakes and streams contained 8–9 species each. Canoe canals were the least preferred habitat (3 species recorded) (Table 3). *Cs*. *inornata* and *Cx*. *stigmatosoma* were the only species present in all habitats (Table 3, S4 and S6 Figs).

**Table 3:** Type of sampling habitat and number of records for each specie.

*Habitat*	*Specie*	*Records*
Canoe canal	***Cs*. *(Cs*.*) inornata***	1
	*Cx*. *(Cx*.*) salinarius*	1
	***Cx*. *(Cx*.*) stigmatosoma***	1
Cemetery	*Ae*. *(Och*.*) epactius*	170
	*Cx*. *(Cx*.*) pipiens quinquefasciatus*	125
	***Cx*. *(Cx*.*) stigmatosoma***	121
	*Cs*. *(Cs*.*) particeps*	108
	*Cx*. *(Cx*.*) pinarocampa*	50
	*Cx*. *(Cx*.*) thriambus*	35
	***Cs*. *(Cs*.*) inornata***	16
	*Cx*. *(Cx*.*) tarsalis*	11
	*Cx*. *(Cx*.*) arizonensis*	9
	*Cx*. *(Cx*.*) bidens*	7
	*Cx*. *(Cx*.*) salinarius*	6
	*Lut*. *(Lut*.*) bigoti*	2
	*Cx*. *(Cx*.*) restuans*	1
	*Cx*. *(Cx*.*) erythrothorax*	1
City Rain Collector	*Cs*. *(Cs*.*) particeps*	9
	***Cx*. *(Cx*.*) stigmatosoma***	9
	*Cx*. *(Cx*.*) pipiens quinquefasciatus*	4
	***Cs*. *(Cs*.*) inornata***	3
	*Cx*. *(Cx*.*) tarsalis*	3
	*Cx*. *(Cx*.*) erraticus*	2
	*Cx*. *(Cx*.*) salinarius*	2
	*Cx*. *(Cx*.*) arizonensis*	1
	*Cx*. *(Cx*.*) pinarocampa*	1
	*Lut*. *(Lut*.*) bigoti*	1
Natural lake	***Cs*. *(Cs*.*) inornata***	6
	***Cx*. *(Cx*.*) stigmatosoma***	5
	*Cx*. *(Cx*.*) tarsalis*	5
	*Cx*. *(Cx*.*) salinarius*	4
	*Cx*. *(Cx*.*) pipiens quinquefasciatus*	2
	*Ur*. *(Ur*.*) sapphirina*	2
	*An*. *(An*.*) aztecus*	1
	*Cs*. *(Cs*.*) particeps*	1
Pond	*Ae*. *(Och*.*) epactius*	3
	*Cx*. *(Nx*.*) apicalis*	3
	*Cs*. *(Cs*.*) particeps*	2
	*Cx*. *(Cx*.*) arizonensis*	2
	***Cs*. *(Cs*.*) inornata***	1
	*Cx*. *(Cx*.*) coronstor*	1
	*Cx*. *(Cx*.*) erraticus*	1
	*Cx*. *(Cx*.*) pipiens quinquefasciatus*	1
	***Cx*. *(Cx*.*) stigmatosoma***	1
Stream	*Cx*. *(Cx*.*) arizonensis*	6
	*An*. *(An*.*) aztecus*	5
	*Cs*. *(Cs*.*) particeps*	4
	*An*. *(An*.*) punctipennis*	1
	***Cs*. *(Cs*.*) inornata***	1
	*Cx*. *(Cx*.*) erythrothorax*	1
	*Cx*. *(Cx*.*) salinarius*	1
	***Cx*. *(Cx*.*) stigmatosoma***	1
Temporarily pond	*Ae*. *(Och*.*) epactius*	50
	*Cs*. *(Cs*.*) particeps*	12
	***Cs*. *(Cs*.*) inornata***	11
	***Cx*. *(Cx*.*) stigmatosoma***	10
	*Cx*. *(Cx*.*) pipiens quinquefasciatus*	8
	*Cx*. *(Cx*.*) tarsalis*	7
	*Cx*. *(Cx*.*) salinarius*	4
	*Cx*. *(Cx*.*) thriambus*	4
	*An*. *(An*.*) aztecus*	2
	*Cx*. *(Cx*.*) coronstor*	2
	*Cx*. *(Cx*.*) pinarocampa*	2
	*Ae*. *(Och*.*) scapularis*	1
	*Cx*. *(Cx*.*) arizonensis*	1
	*Cx*. *(Cx*.*) erythrothorax*	1
	*Cx*. *(Nx*.*) apicalis*	1
Water traffic corridor	*An*. *(An*.*) aztecus*	11
	***Cs*. *(Cs*.*) inornata***	8
	*Cx*. *(Cx*.*) salinarius*	8
	***Cx*. *(Cx*.*) stigmatosoma***	8
	*Cx*. *(Cx*.*) tarsalis*	7
	*Cx*. *(Cx*.*) pipiens quinquefasciatus*	4
	*Cx*. *(Cx*.*) erythrothorax*	3
	*Ae*. *(Och*.*) epactius*	3
	*Cs*. *(Cs*.*) particeps*	2
	*Cx*. *(Nx*.*) apicalis*	2
	*Ur*. *(Ur*.*) sapphirina*	2
	*An*. *(An*.*) punctipennis*	1
	*Cx*. *(Cx*.*) erraticus*	1
	*Ur*. *(Ur*.*) lowii*	1
	*Lut*. *(Lut*.*) bigoti*	1
Wetland	*An*. *(An*.*) aztecus*	5
	*Cx*. *(Cx*.*) salinarius*	3
	***Cs*. *(Cs*.*) inornata***	2
	*Cs*. *(Cs*.*) particeps*	2
	***Cx*. *(Cx*.*) stigmatosoma***	2
	*Cx*. *(Cx*.*) tarsalis*	2
	*Cx*. *(Nx*.*) apicalis*	2
	*An*. *(An*.*) punctipennis*	1
	*Cx*. *(Cx*.*) erythrothorax*	1
	*Ur*. *(Ur*.*) sapphirina*	1

*Cs*. *inornata* and *Cx*. *stigmatosoma* (in bold) were recorded in all habitats.

The presence of *Ae*. *aegypti* was detected over three consecutive years (2015–2017) using ovitraps (Table 4, Fig 3). Occurrence was detected predominantly in the warmer microclimate (5°C—28°C: in the north of the city) (Tables 2 and 4). Although each year has seen increases in the number of positive ovitraps, at the sites have not been consistent, with the exception of “Tapo” Bus Terminal (19^o^ 25’ 44” N, 99^o^ 06’46” W), where the occurrence of *Ae*. *aegypti* was recorded for two consecutive years (2016–2017) (Table 4). The average number of eggs laid in each ovitrap was 21.33, where the percentage of *Ae*. *aegypti* eggs hatched was 8 (37%).

**Table 4:** Location of *Aedes aegypti* and *Aedes albopictus* eggs collected by ovitraps in Mexico city.

*Year*	*Positive ovitrap site*	*Description*	*Min-max climate range in Mexico City*	*Latitude (N)*	*Longitude (W)*
***Aedes aegypti***					
2015	Casa del Peregrino	Pilgrim house	5°C—28°C	19^o^ 28’ 57”	99^o^ 06’ 37”
	Estación Pantaco	Train station	5°C—28°C	19^o^ 28’ 59”	99^o^ 10’ 09”
2016	CONALEP Aeropuerto	Voc-Tech high school	5°C—28°C	19 ^o^ 25’ 26”	99^o^ 03’ 26”
	TAPO	Bus terminal	5°C—28°C	19^o^ 25’ 44”	99^o^ 06’ 46”
	Alameda Oriente	Public park	5°C—28°C	19^o^ 26’ 08”	99^o^ 03’ 08”
2017	Parque Santa Úrsula	Public park	4°C—26°C	19^o^ 18’ 12”	99^o^ 09’ 26”
	Museo Diego Rivera Anahuacalli	Museum	4°C—26°C	19^o^ 19’ 22”	99^o^ 08’ 38”
	Alberca Salvador Allende	Community swimming pool	5°C—28°C	19^o^ 21’ 29”	99^o^ 03’ 02”
	Parque del Pueblo Cuitláhuac	Farm park & zoo	5°C—28°C	19^o^ 21’ 41”	99^o^ 02’ 38”
	Central de Abastos	Food supply center	5°C—28°C	19^o^ 22’ 45”	99^o^ 05’ 35”
	Deportivo Venustiano Carranza	Community sport center	5°C—28°C	19^o^ 25’ 35”	99^o^ 07’ 06”
	TAPO	Bus terminal	5°C—28°C	19^o^ 25’ 44”	99^o^ 06’ 46”
	Gimnasio “Smart Fit” Molina	Gym parking lot	5°C—28°C	19^o^ 29’ 51”	99^o^ 05’ 23”
	Parque Nacional El Tepeyac	National Park	5°C—28°C	19^o^ 30’ 14”	99^o^ 06’ 28”
	Panteón Ticomán	Cemetery	5°C—28°C	19^o^ 30’ 18”	99^o^ 07’ 16”
	Deportivo Atlético Mexicano	Community sport center	5°C—28°C	19^o^ 22’ 29”	99^o^ 03’ 46”
	Zoológico de San Juan de Aragón	Zoo park	5°C—28°C	19^o^ 27’ 43”	99^o^ 05’ 02”
	Jardín Francisco J. Múgica	Public park	5°C—28°C	19^o^ 20’ 57”	99^o^ 03’ 41”
***Aedes albopictus***					
2017	Cerro de la Estrella	National Archeological Park	5°C—28°C	19^o^ 21’ 06”	99^o^ 05’ 30”
	Parque Nacional El Tepeyac	National Park	5°C—28°C	19^o^ 29’ 58”	99^o^ 06’ 23”

For the first time, *Ae*. *albopictus* was detected in the city. In 2017, *Ae*. *albopictus* were detected in two sites in the warm northern region of Mexico City in national parks (“El Tepeyac” and “Cerro de la Estrella”) (Table 4, Fig 3). At the “El Tepeyac” park, 125 eggs were collected, but only two hatched larvae were identified as *Ae*. *albopictus*. At “Cerro de la Estrella” park, 27 eggs were collected, but only one larva was recognized as *Ae*. *albopictus*.

**Fig 4:**
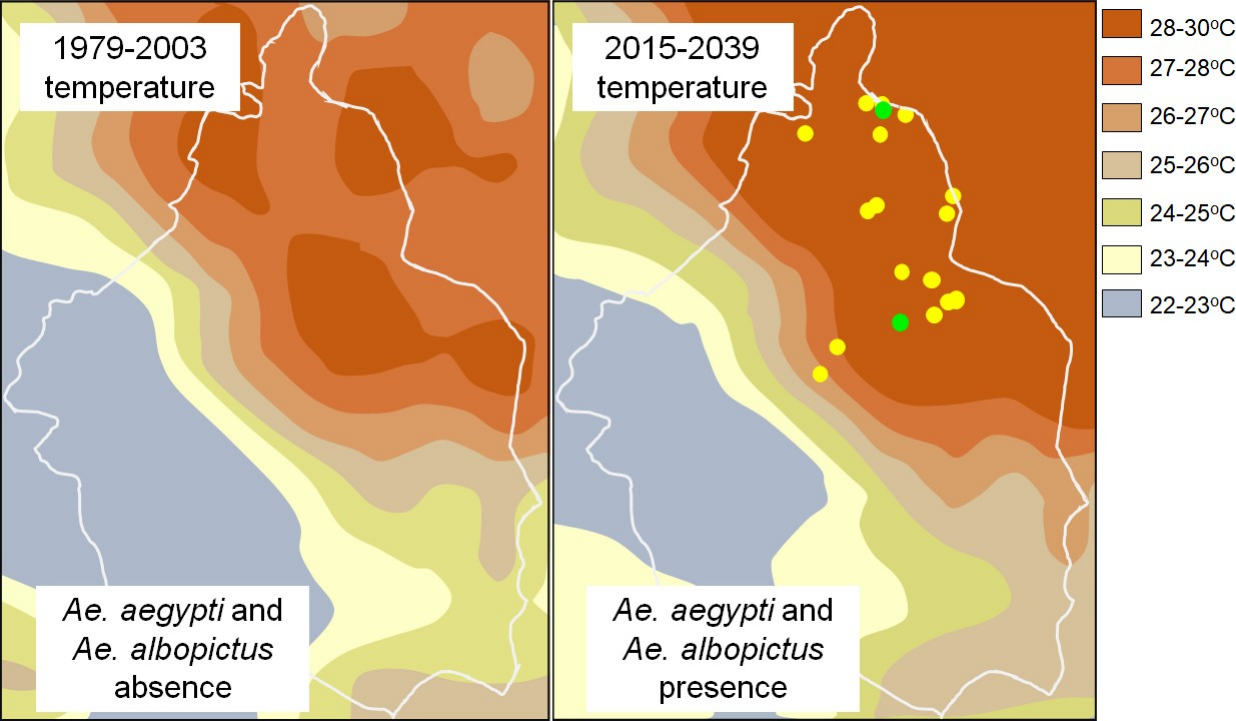
Possible correlation between the projected increase in temperatures (modified from [20]) and the current presence of *Ae*. *aegypti* (yellow dots) *and Ae*. *albopictus* (green dots) in the city.

When overlaying *Ae*. *aegypti* and *Ae*. *albopictus* distribution on the temperature projection, collection sites and predicted warmer temperatures areas were highly correlated (Fig 4). These zones might provide climatic and habitat suitability which could promote the long-term establishment of the species.

## Discussion

The updated list now includes a total of 26 mosquito species (excluding *Cx*. *peus* and *Cs*. *Dugesi*, because synonymy) in Mexico City. Nine new records, and the intermittent but increased presence of *Ae*. *aegypti*, were documented. The present surveillance effort was the first to sample mosquitoes over a large extension of the city. Since previous collections may not have surveyed all the water bodies as in our present study, the newly recorded species may have been historically present.

New records include: *Ae*. *scapularis*, a common species in Mexico, mainly with neotropical distribution; *An*. *punctipennis* and *Cx*. *erraticus*, nearctic common species with some neotropical distribution; *Cx*. *bidens*, a species with nearctic and neotropical distribution; *Lut*. *bigoti*, mainly a neotropical species, distributed in the Balsas Basin and South Pacific Zone; *Ur*. *lowii*, a neotropical common species with some nearctic distribution [7,10].

A particularly interesting new record was *Ae*. *epactius*. Its presence, but mostly its great abundance and distribution, was surprising given that there were no previous records for this species in the city. Although Diaz-Badillo *et al*. [8] reported the presence of *Ochlerotatus spp*, identification was not made at the species level. *Ae*. *epactius* is a common species in the southern United States and Mexico [10,17] at low, mid and high elevations [39]. Lozano-Fuentes *et al*. [39] had speculated that a possible reason for increased distribution at higher elevations could be climate change. The lack of previous records of this species in the city may be explained by a possible event of recent colonization. The presence of *Cx*. *pinarocampa* was another interesting finding. Previous records only reported this species from five states of Mexico (Oaxaca, Chiapas, Veracruz, Guerrero and Estado de Mexico) [7]. This species occurred in 29% of our collecting sites, however, the number of collected individuals was low.

### Microclimates and clusters

Microclimates one-two, 1–3 and 2–3 shared several species (Jaccard index = 0.31, 0.33 and 0.31 respectively). It could be argued that the continued increase in temperature can explain this pattern. High urban temperatures may have been promoting the expansion of species ranges (see Jauregui 1997), with, some species now adapted to a wide range of temperatures. For the case of *Cs*. *particeps* (distributed in the four microclimates), the increasing temperatures could be changing their distribution in the city.

Microclimate two (4°C—26°C), showed 22 species, eight were exclusively found here and four of these were confined to cluster five (spot D “Xochimilco”). The “Xochimilco” area is set of canals and wetlands, both remnants of the extinct large saline Lake of Xochimilco [40]. In 1987, the area was included on UNESCO’s World Heritage List [41]. However, previous high deforestation rates, rapid ongoing illegal urbanization and overexploitation of groundwater have caused a significant reduction of the wetland area [42]. For the four species that were only found here, a process of intense local adaptation could had caused their restricted distribution.

Habitat loss may have also promoted the isolation of populations. For example, *Cx*. *erraticus Ae*. *scapularis*, *Cx*. *apicalis*, *Cx coronator* and *Cx erythrothorax* were only present in ecological preserves (Pedregal: 19^o^19’06” N, 99^o^11’04” W and Xochimilco) and/or well-preserved fragmented areas (Milpa Alta, cluster E). The same could be occurring for some species found in clusters four and six. Current trends in urbanization may threaten the persistence of native mosquitos in the city.

Clusters “A, B and C” were completely immersed in urban areas and showed high larval productivity. In this area there is an assemblage of untreated habitats, useful for both possible vectors and/or nuisance mosquitoes. Clusters D and E included ecological or national parks, surrounded by residential or industrial areas. The bordering urban sites may provide natural and artificial containers that could be used as larval habitats. They could also be transition areas between sylvatic and truly urban zones.

### Habitats

The abundance of larvae appeared to be most influenced by habitat type. For *Aedes* and *Culex* spp. [43,44], cemeteries have long been recognized as major breeding sites with suitable conditions for larval development (abundant containers, vegetation cover and landscape connectivity). For these reasons, they have been under constant monitoring. As expected, cemeteries showed the greatest productivity and diversity of mosquitoes (14 species, 10 belonging to the *Culex* genus). *Ae*. *epactius*, *Cx*. *p*. *quinquefasciatus* and *Cx*. *stigmatosoma*, were the most frequent observed species. Cemeteries should remain as priority sites for the monitoring of mosquitoes.

Mexico City is a heterogeneous mosaic of residential and commercial areas, parks, and other land-use types. This also provides an array of temporary pools that are being frequently used by mosquitoes. Although temporary pools could also be in the same areas as cemeteries, the edaphic conditions and assemblage of predators and prey may be completely different. This could help explain the differences related to species assemblage between cemeteries and temporary pools.

Rain collectors were located in the Milpa Alta borough, in microclimate three. Meanwhile, two streams were situated in microclimate four and one stream in the Xochimilco area. Milpa Alta and microclimate four were the less urbanized and colder zones. These areas could provide harborage and dispersal routes for sylvatic/native species.

Wetlands, water corridors, natural lakes and a pond are part of the remnants of the Xochimilco and Chalco extinct saline lakes (located in the southwestern part of the urban area of the city). These water bodies have alkaline pH and a high content of organic matter [45,46]. Interestingly, regardless of its size and freshwater capacity (470, 625 m^3^; [47]), canoe canals harbored only three species: *Cx*. *salinarius*, *Cs*. *inornata* and *Cx*. *stigmatosoma*. Pollution could be an important consideration in the canal. Algae and cyanobacteria toxicity blooms are constant [47,48], limiting habitat suitability for some species. The salinity, pollution and pH of these habitats could explain the presence of few species and the low abundance of species like *Ae*. *epactius* and *Cx*. *p*. *quinquefasciatus*. In our study, *Cs*. *inornata* and *Cx*. *stigmatosoma* were the only species capable of exploiting all available habits. It is unknown if larvae of both species are adapting to polluted sites or to man-made environments.

### *Aedes aegypti* and *Aedes albopictus* presence: Risks and challenges

Our study created a starting point for future efforts aimed to addressing *Ae*. *aegypti* and *Ae*. *albopictus* distribution across the city. The presence of *Ae*. *aegypti* has significantly increased. Unsurprisingly, positive ovitrap collections were recorded in the warmer areas, in the north and northeast of the city. The first records of *Ae*. *aegypti* in 2015 occurred in a train station and a pilgrim house. Both sites are places that experience massive local and non-local human transit. We presume that an accidental introduction of eggs, larvae, pupae, and/or adults into the region occurred by human transit [4]. However, the 2016–2017 collections were recorded at public parks, museums, residential areas and cemeteries, indicating the possible presence of transitory colonies. To date, it remains unclear if true colonization has occurred since no larvae or adults have been collected.

*Ae*. *albopictus* was only collected at the warmer area of the city. The National park “El Tepeyac” is next to the Basilica of Guadalupe, one of the most important pilgrimage sites of Catholicism in the Americas which is annually visited by millions of people from different states and countries. The National Park “Cerro de la Estrella”, and its surroundings, are the venues for the Iztapalapa Catholic Passion Play (a representation of the crucifixion of Jesus). Annually, almost 2 million persons (local and non-local) gather to observe the play [49]. Given the high level of transit into the parks from areas with endemic *Ae*. *albopictus*, independent introductions of adults or immature stages are likely to occur. However, it also remains unclear if colonization has occurred.

Temperature and habitat availability are two important factors affecting the presence of mosquitoes. Climate change might be a causative factor for introduction of *Ae*. *aegypti* [13], especially in urban areas, given that water and air temperature in urban areas are higher than in suburban ones [50]. Reports of *Ae*. *aegypti* being in areas either with elevated altitudes and/or cold temperatures show that migration and colonization in colder areas seems to be becoming more common [51–53]. Deforestation, poor housing and insufficient sewer and waste management systems are consequences of uncontrolled urbanization. All these factors could be leading to the increase of suitable habitats for *Aedes spp*. However, not all water bodies in the city are necessarily at risk; for example, the Xochimilco area includes turbid, saline or polluted breeding sites where *Aedes* larvae are not likely to occur.

Historically, the altitude and geographical location of Mexico City could have limited *Ae*. *albopictus and Ae*. *aegypti* presence. Increasing levels of urbanization put Mexico City and neighboring areas at risk of becoming areas where vector mosquitoes (and the diseases they carry) could become established. Because of their close association with humans, surveillance for larval and adult *Ae*. *aegypti* and *Ae*. *albopictus* should occur near human dwellings, schools and residential/commercial areas (F. Castelo, pers.comm). Cemeteries should also be priority sites for monitoring and mosquito control [54].

Cemeteries seem to be the most suitable habitats for *Ae*. *aegypti* and *Ae*. *albopictus* to colonize. However, we have detected the presence of multiple other species in these habitats. Competition among larvae is an important factor regulating mosquito populations [55]. *Aedes* spp are known to alter competitive interactions, declining the presence of other species [14]. Nevertheless, it is also possible that native species could influence the population growth of invasive larvae, imposing barriers to a successful invasion. The potential impact of species competition among native species and *Ae*. *aegypti* should be evaluated.

Competition and climate change may also impact adult mosquito susceptibility to arboviral infections [56]. Since vector borne diseases can also be influenced by climate, extreme weather may impact the presence of several infectious diseases. It is possible that warmer temperatures could trigger the introduction of pathogens. It has been proposed that climate change will contribute to an extensive increase in the number of people at risk of dengue fever [57], although recent findings suggest a low potential for ZIKV transmission at high elevations [58]. Twenty-three species reported here have medical importance. Consequently, a constant surveillance of arboviral diseases and vectors must be a priority in the city

## Conclusions

Our findings provide a starting point to create a suitable plan for mosquito control in Mexico City. A deeper understanding of the spatio-temporal dynamics of breeding sites and microecological habitat characteristics in Mexico City is required. Identifying associations between biological diversity and habitats may us enable to predict how populations will respond to habitat reduction, species competition and climate change.

Currently, *Ae*. *aegypti* and *Ae*. *albopictus* do not seem to be established in Mexico City, however, they were included in the updated list because of their rapid and successful colonizing abilities. The presence of the main vector of dengue, chikungunya and Zika shows that Mexico City should no longer be considered exempt from the occurrence of vector-borne disease outbreaks. Thus, the practice of arbovirus control through community participation, mass media and education programmes in schools should be introduced.

## Supporting information

S1 Fig *Aedes aegypti* and A*e. albopictus* collecting sites. All sites were peri-domestic areas, including: cemeteries, public (free access) parks, museums or community centers.
(TIF)

S2 Fig Distribution of *Ae. epactius, Ae. scapularis, An. Aztecus* and *An. punctipennis* of Mexico City collected in the present study. (TIF)

S3 Fig Distribution of *Cx. bidens, Cx. coronator, Cx. erythrothorax* and *Cx. pinarocampa* of Mexico City collected in the present study. (TIF)

S4 Fig Distribution of *Cx. restuans, Cx. salinarius, Cx. stigmatosoma* and *Cx. tarsalis* of Mexico City collected in the present study. (TIF)

S5 Fig Distribution of *Cx. thriambus, Cx. quinquefasciatus, Cx. erraticus* and *Cx. apicalis* of Mexico City collected in the present study. (TIF)

S6 Fig Distribution of *Cs. ionoranta, Cs. particeps, Lut. bigoti* and *Ur. lowii* of Mexico City collected in the present study. (TIF)

S7 Fig Distribution of *Ur. sapphrina* of Mexico City collected in the present study. (TIF)
