# Supplementary figures and images for: Urban and semi-urban mosquitoes of Mexico City: A risk for endemic mosquito-borne disease transmission
Source: PLoS One. 2019 Mar 6;14(3):e0212987. doi: 10.1371/journal.pone.0212987 (PMC6402764; doi:10.1371/journal.pone.0212987)

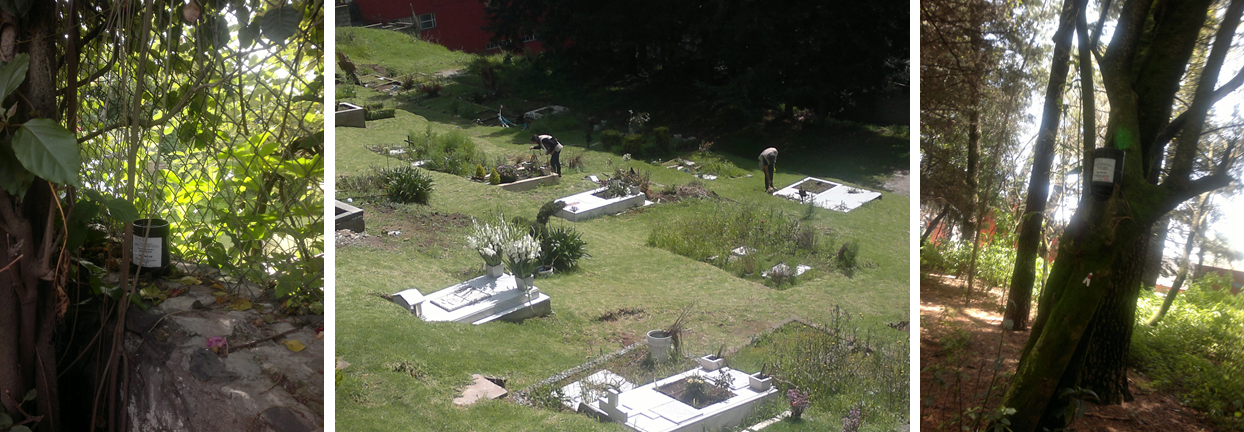

Supplement: S1 Fig — All sites were peri-domestic areas, including: cemeteries, public (free access) parks, museums or community centers. (TIF) [file pone.0212987.s001.tif]

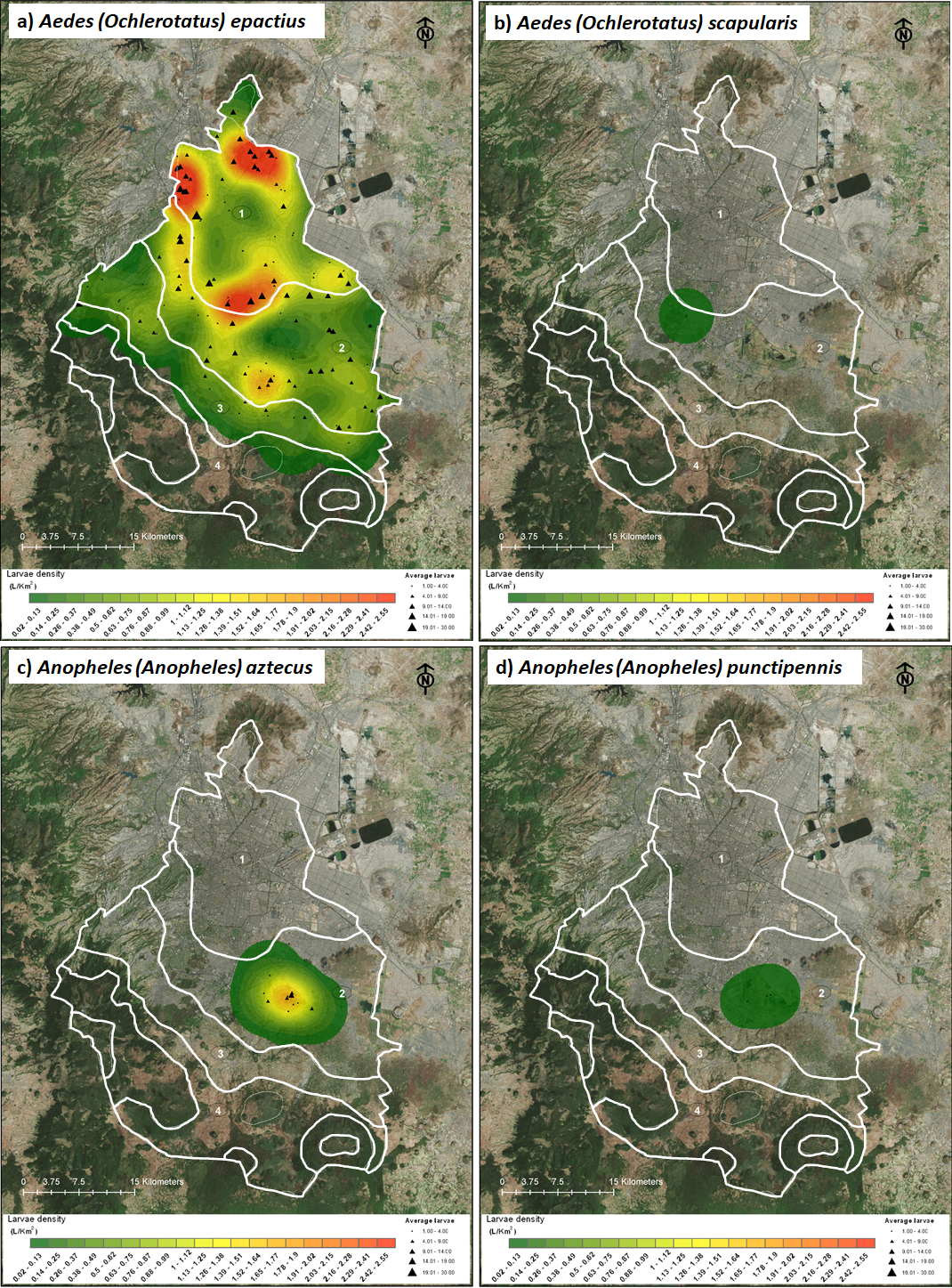

Supplement: S2 Fig — (TIF) [file pone.0212987.s002.tif]

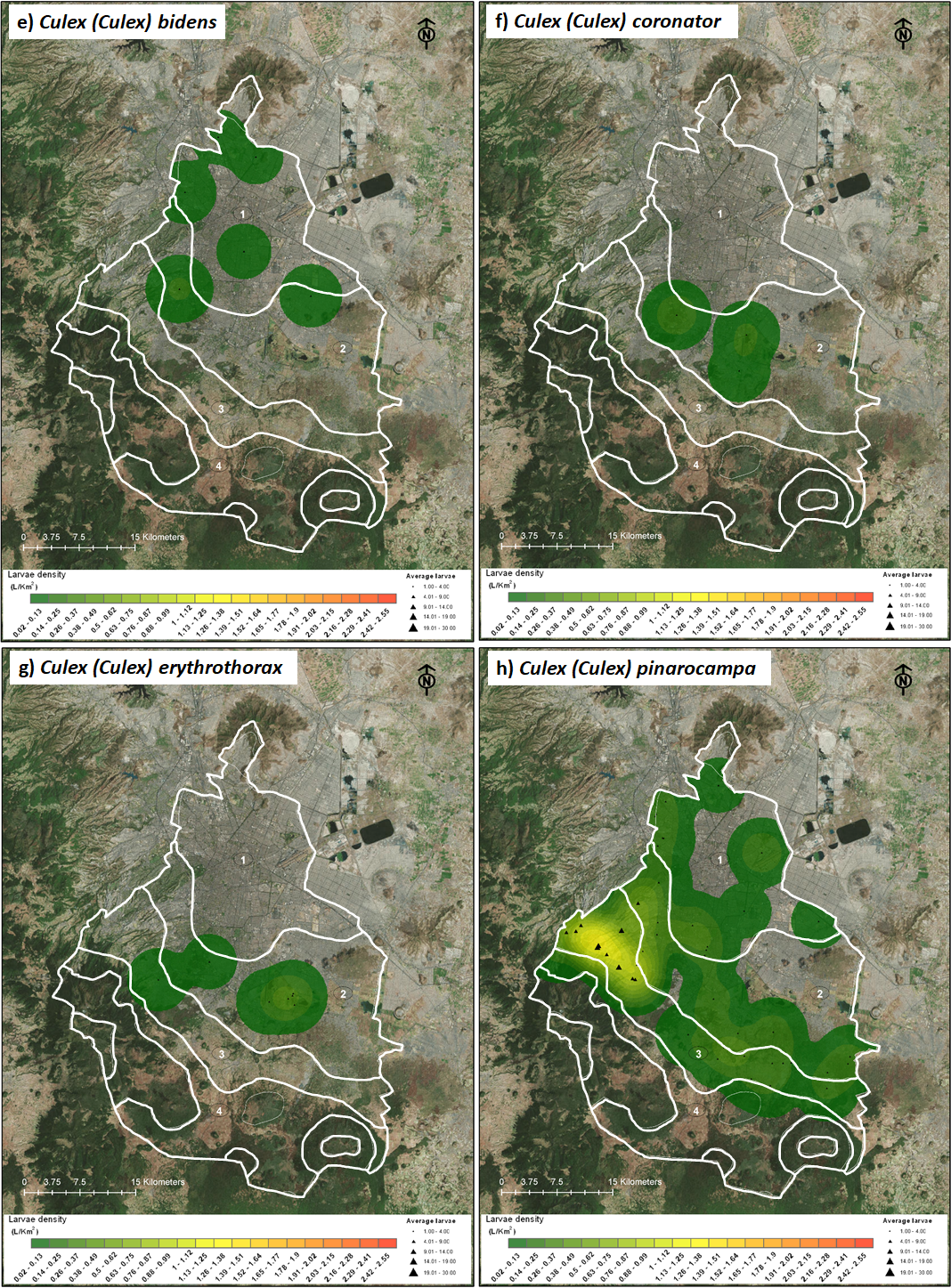

Supplement: S3 Fig — (TIF) [file pone.0212987.s003.tif]

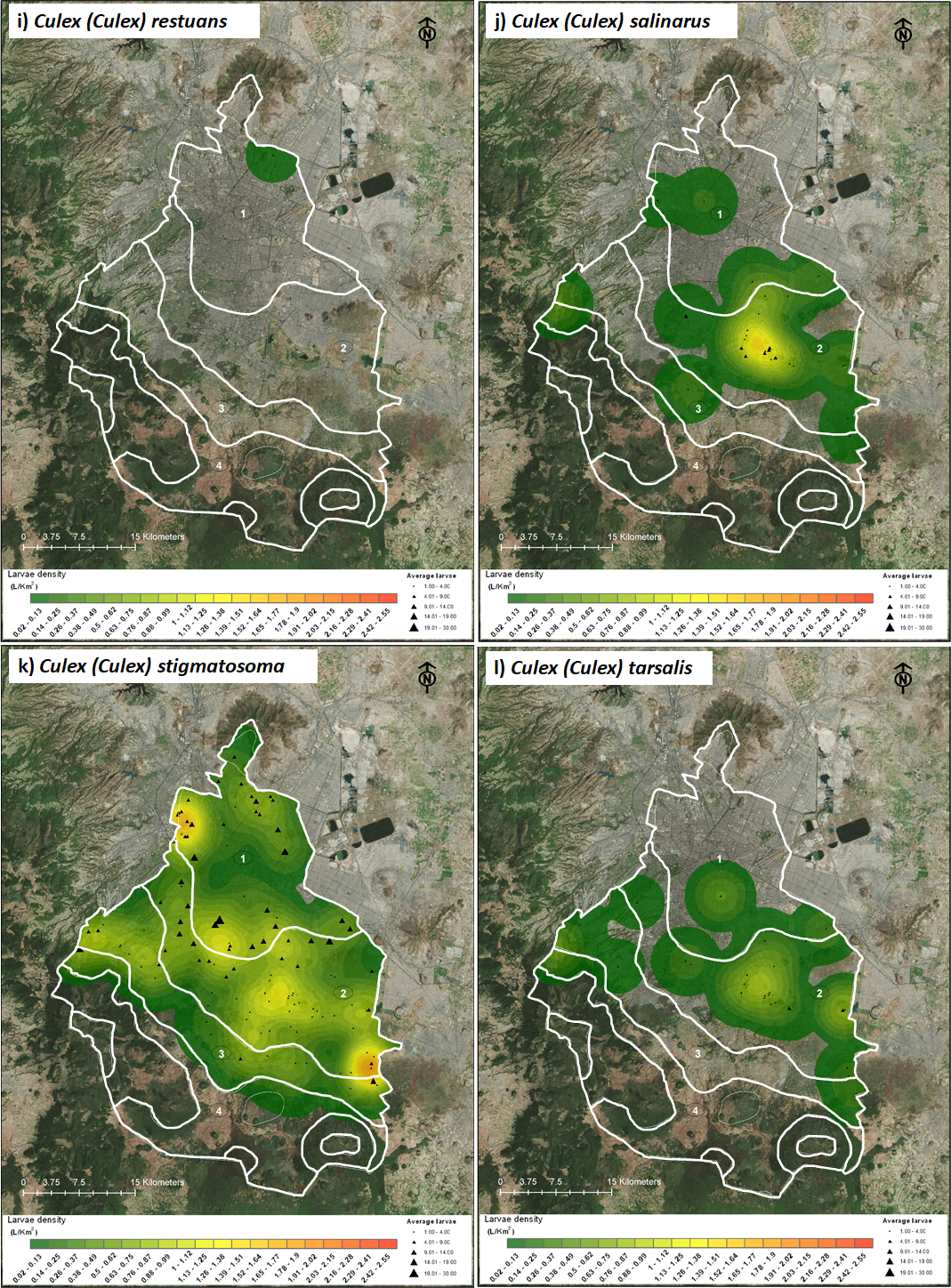

Supplement: S4 Fig — (TIF) [file pone.0212987.s004.tif]

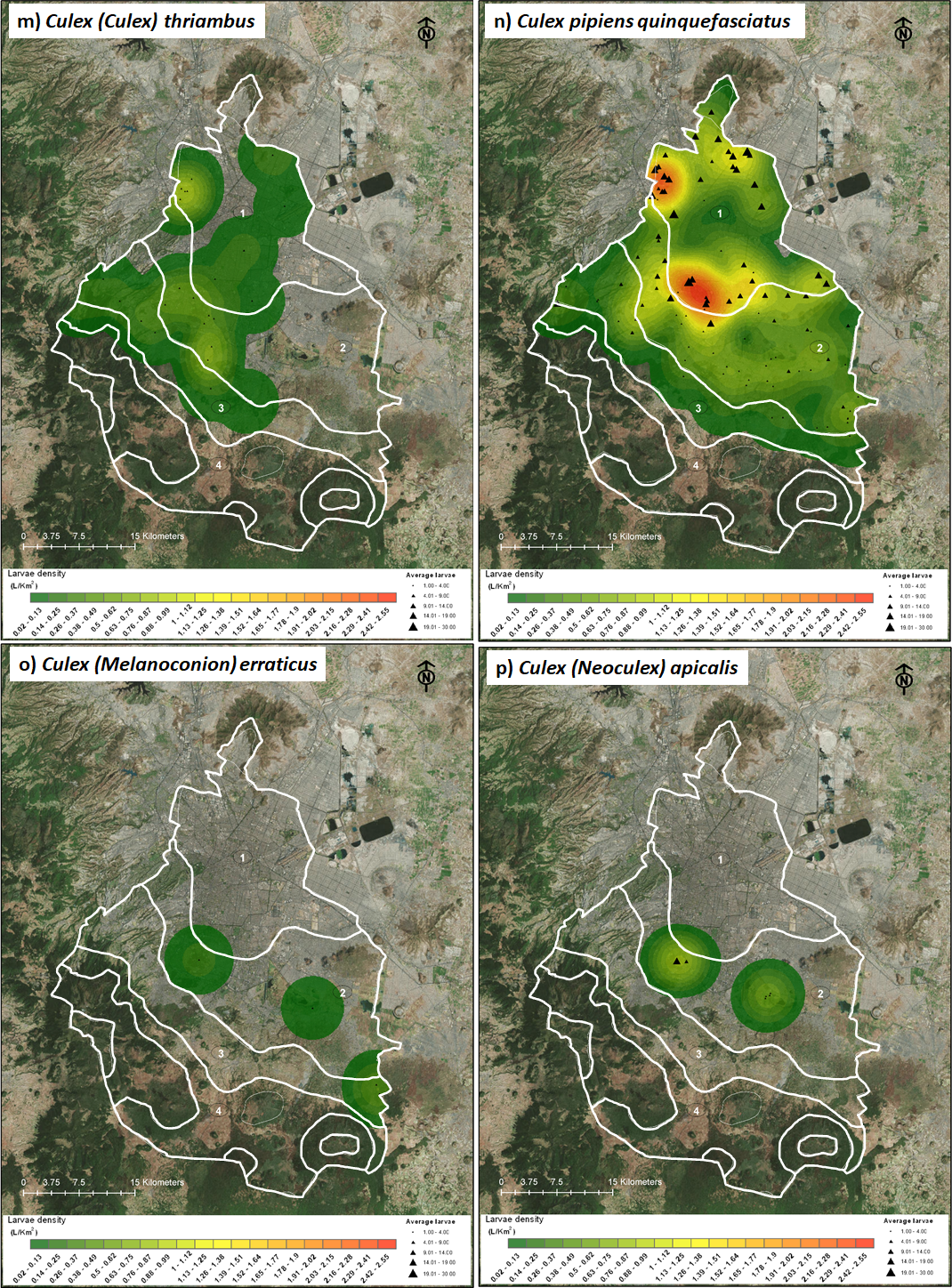

Supplement: S5 Fig — (TIF) [file pone.0212987.s005.tif]

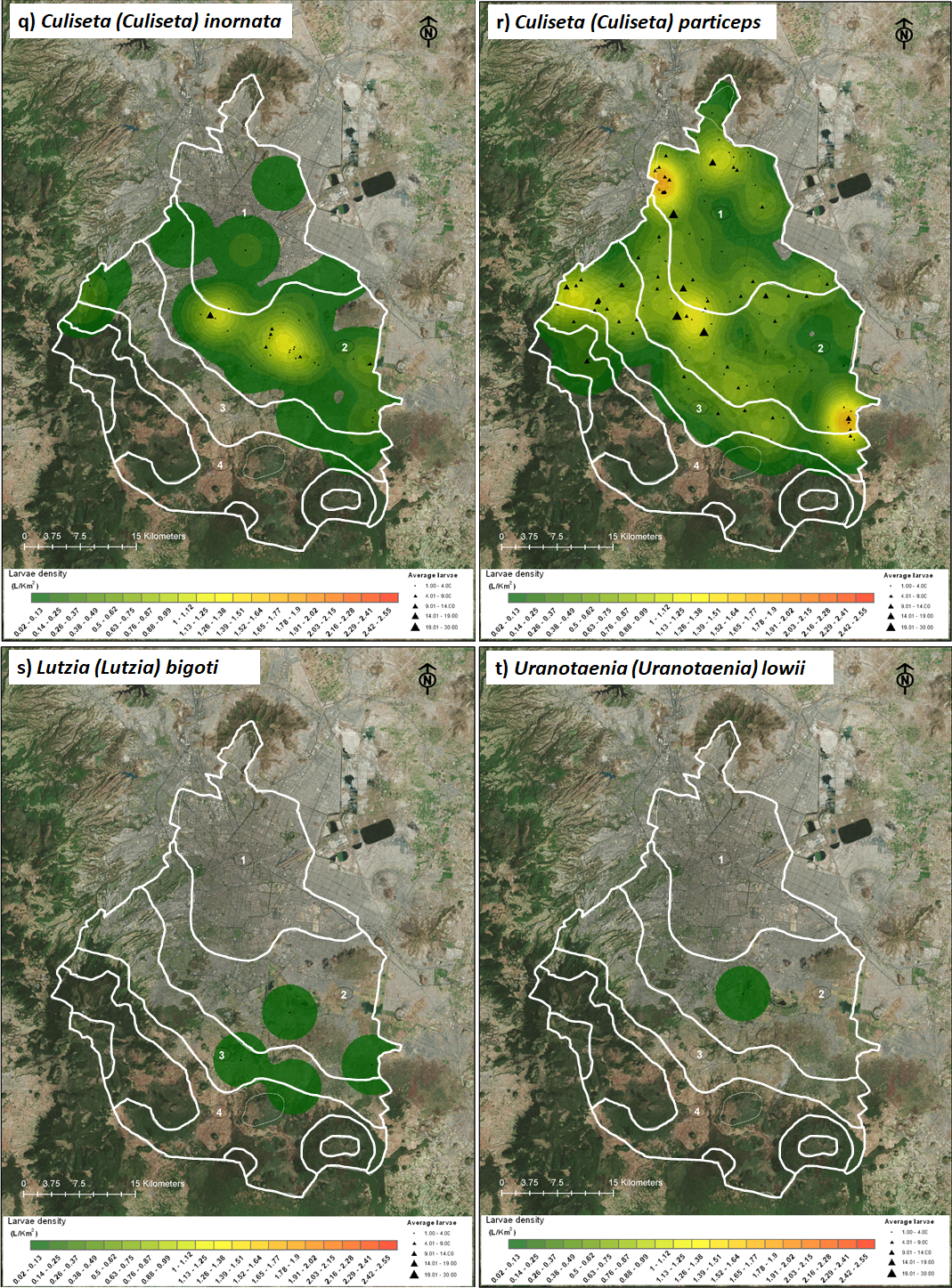

Supplement: S6 Fig — (TIF) [file pone.0212987.s006.tif]

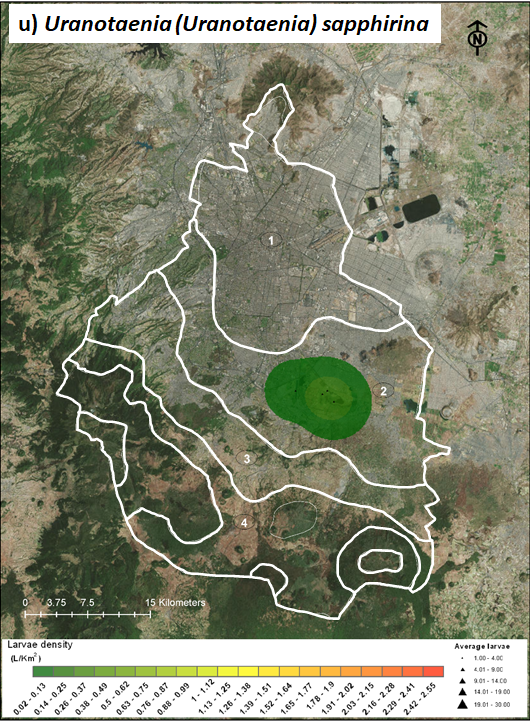

Supplement: S7 Fig — (TIF) [file pone.0212987.s007.tif]
